# Cytokine-induced killer cells as a feasible adoptive immunotherapy for the treatment of lung cancer

**DOI:** 10.1038/s41419-018-0404-5

**Published:** 2018-03-06

**Authors:** Dan Chen, Huanhuan Sha, Tianmu Hu, Shuchen Dong, Junying Zhang, Siwen Liu, Haixia Cao, Rong Ma, Yang Wu, Changwen Jing, Zhuo Wang, Jianzhong Wu, Jifeng Feng

**Affiliations:** 1 Research Center of Clinical Oncology, Jiangsu Cancer Hospital, Jiangsu Institute of Cancer Research, Nanjing Medical University Affiliated Cancer Hospital, Nanjing, China; 2 The Forth Clinical School of Nanjing Medical University, Nanjing, China; 3 Department of Biological Science, Purdue University, West Lafayette, IN USA

## Abstract

Most of the patients with lung cancer are diagnosed at advanced stage, and they often lose the opportunity of surgical therapy, most of whom fail to reach good prognosis after chemotherapy. Recently, a few clinical studies have confirmed the role of adoptive T-cell transfer in the maintenance therapy of cancer patients. Here, we provided statistical insights into the role of CIKs in advanced lung cancer from three different levels, cell model (in vitro co-culture system), mice model (in situ lung cancer), and clinical research (in lung cancer patients of different progression stages). We optimized the components of supplements and cytokines on activating and expanding CIK cells. Based on this, we explored a new serum-free medium for in vitro activation and expansion of CIK cells. Moreover, we found that activated CIK cells could efficiently kill lung cancer cells in cell-to-cell model in vitro and significantly reduce the tumor growth in mice. For the clinical research, the OS rates of patients received combination of chemotherapy and CIK treatment were significantly improved compared to the OS rates of patients only received chemotherapy. Additionally, CIK therapy represented good toleration in our study. All the results suggested that combination of immunotherapy with traditional therapy will be a feasible and promising method for the treatment of lung cancer.

## Introduction

The morbidity and mortality of lung cancer have increased rapidly in recent years, with the 5-year survival rate of only ~15%. About 80–85% of lung malignancies are non-small cell lung cancer (NSCLC). Most NSCLC patients are diagnosed at advanced stage, which deprive the opportunity of timely surgical therapy. The delays in diagnosing develops to disease progression in long term and poor overall survival (OS). Epidermal growth factor receptor-tyrosine kinase inhibitor (EGFR-TKI) is effective in NSCLC patients carrying sensitive EGFR mutations^1^. Nevertheless, prolonged cancer treatment with TKI will induce the development of acquired resistance to TKI within 8–14 months^2,3^. Therefore, developing a new therapy method is necessary to reduce the side effect of chemotherapy and to improve the OS in NSCLC patients.

Cancer immunotherapy is the fourth cancer treatment technology besides surgery, chemotherapy, and radiotherapy^4–7^. Different from the other three therapies, cancer immunotherapy focuses on improving anti-cancer abilities of immune cells rather than killing cancer cells directly^8–10^. Currently, cancer immunotherapy includes immune checkpoint inhibitor therapy, adoptive immunotherapy, engineered T-lymphocyte-based cell therapy, immunomodulatory drugs, and cancer vaccine^11,12^. One potential alternative to reconstitute host immunity is adoptive immunotherapy, which can eliminate cancer cells through transfusing in vitro expanded and activated immune cells, such as cytokine-induced killers (CIKs)^13–16^, natural killers (NKs)^17,18^, cytotoxic lymphocytes (CTLs), and tumor-infiltrating lymphocytes (TILs)^19–21^.

Autologous CIK cells were activated and expanded from the patients’ peripheral blood mononuclear cells (PBMCs) ex vivo and then were transfused back to the patients^14,22^. CIK cells, also called NKT (T cells with NK phenotype), can be activated and expanded up to 200- to 1000-fold in 14–21 days of culture after initial priming with CD3 antibodies and a set of cytokines^16,23^. Ex vivo-expanded CIKs are a group of CD3^+^ CD56^+^ cells and show potent cytotoxic activity against a number of tumor cell lines or animal models bearing tumor. Some clinical trials have demonstrated that CIKs immunotherapy-combined chemotherapy has potential benefits compared to chemotherapy alone in patients suffering from advanced NSCLC^22–25^. The benefit of immunotherapy is eliminating cancer cells with enough effective immune cells while leaving healthy cells and tissues untargeted. Recent clinical success has inspired the potential for combination of adoptive cell immunotherapy with traditional therapy to gain potent, effective, and durable clinical responses^14,16,23^.

In the current study, we have optimized the components of supplements and the inserted sequence of cytokines on activating and expanding CIK cells. We have explored a new serum-free medium (SFM) for in vitro activation and expansion of T cells, which can kill the lung cancer cells in vitro co-culture system and protect in situ mice models from lung cancer. In addition, we have retrospected hundreds of clinical cases for CIKs-based immunotherapy. We asked whether combination of CIKs and chemotherapy would be potent to prevent patients from undergoing NSCLC. The results showed that the OS rates of patients received combination of chemotherapy and CIK treatment were significantly improved compared to the OS rates of patients received sole use of chemotherapy. Therefore, combination of immunotherapy with chemotherapy will be an effective and promising method for the treatment of patients with lung cancers.

## Results

### The activation and expansion of CIKs

**Fig. 1:**
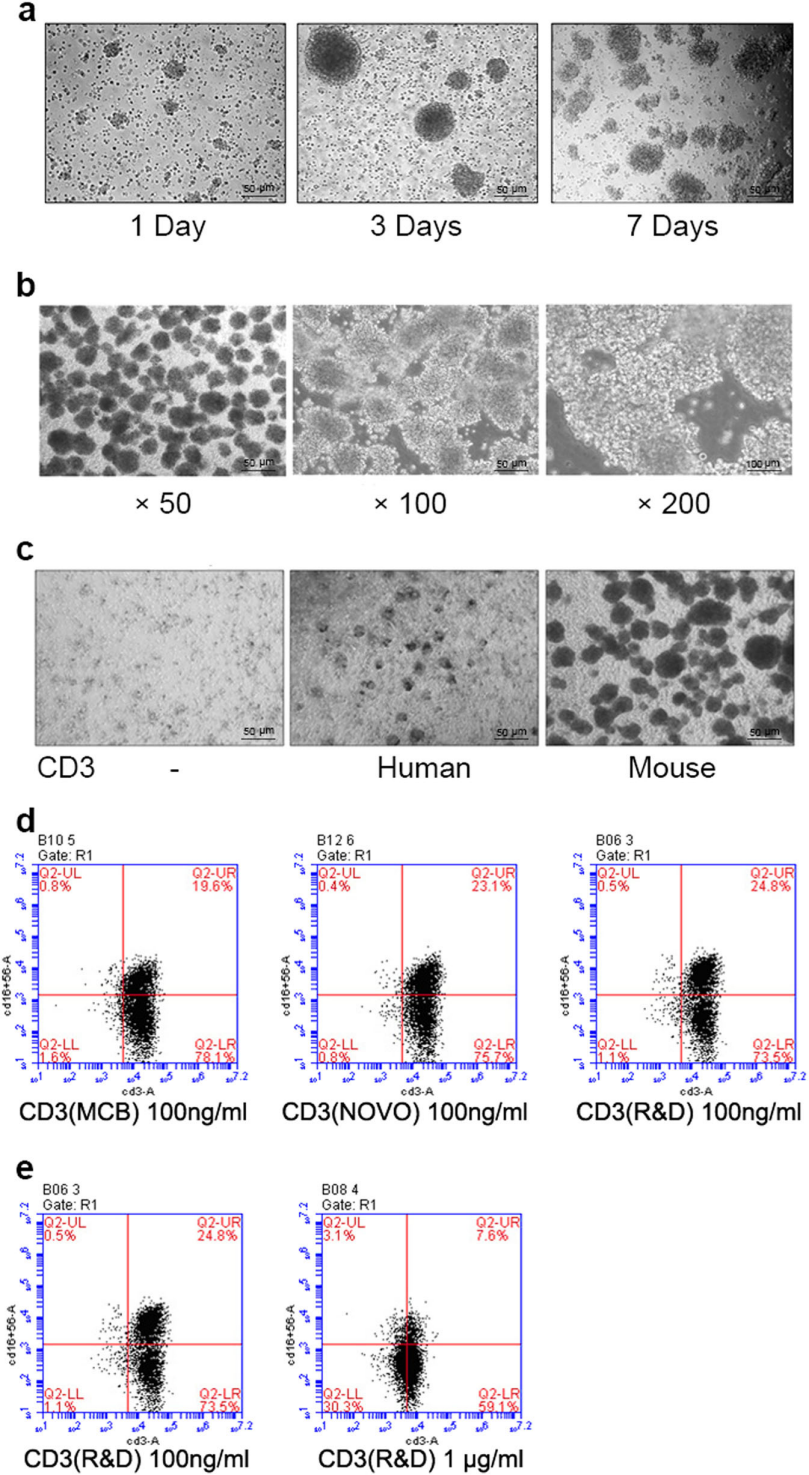
Activation and expansion of CIK cells. **a** Morphology of CIK cells during 7 days of induction. The differentiation and amplification of CIK cells were conducted referring to the Methods. Cells were observed under a microscope on days 1, 3, and 7, respectively (magnification, ×50). **b** Morphology of mature CIK cells. A cluster of CIK cells were observed under a microscope after 14 days of induction (magnification, ×50, ×100, ×200). **c** The function of anti‑CD3 antibody on the activation of CIK cells. Cells were incubated with none, human, or mouse anti-CD3 Abs, and cells were observed under a microscope on days 14 (magnification, ×50). **d** The effects of different brands of anti-CD3 monoclonal antibodies (clone OKT3) on the activation of CIK cells. Cell phenotypes (CD3^+^CD56^+^) were identified by flow cytometry. **e** The effects of different concentration of anti-CD3 mAbs on the activation of CIK cells. Cells were incubated with 100 ng/mL or 1 μg/mL mouse anti-CD3 mAbs during the culture, and cell phenotypes (CD3^+^CD56^+^) were identified by flow cytometry

CIKs derived from PBMCs can grow in suspension and be activated in vitro with anti-CD3 antibody and a set of cytokines (interferon (IFN)-γ, interleukin(IL)-1α, and IL-2). Activated CIKs facilitate aggregation together. Clusters of CIK cells were observed on the third day after activation. The number of clusters had been increasing rapidly during the first 7 days (Fig. 1a). Activated CIKs can aggregate together to form multiple transparent characteristic cell clusters, which were observed at the 14th day under a microscope (magnification, x50, x100, x200) (Fig. 1b). Anti-CD3 antibody was used to proliferate T lymphocytes. The mouse anti‑CD3 antibody (clone OKT3) can effectively enhance the activation and expansion of CIKs, but the human anti-CD3 antibody cannot induce the expansion of CIKs (Fig. 1c). CD3/CD56 double-positive cells represent the activated CIK cells. In addition, different brands of mouse anti-CD3 monoclonal antibodies (clone OKT3) exhibit similar abilities on the enhancement of activation and expansion of CIKs (Fig. 1d). Anti-CD3 mAbs of 100 ng/mL was the sufficient dosage to effectively activate and expand CIKs while no similar effect found for group with a dosage of 1 μg/mL anti-CD3 mAbs (Fig. 1e).

### The effect of the optimal components of supplements and the add sequence of cytokines on the activation and proliferation of CIKs

**Fig. 2:**
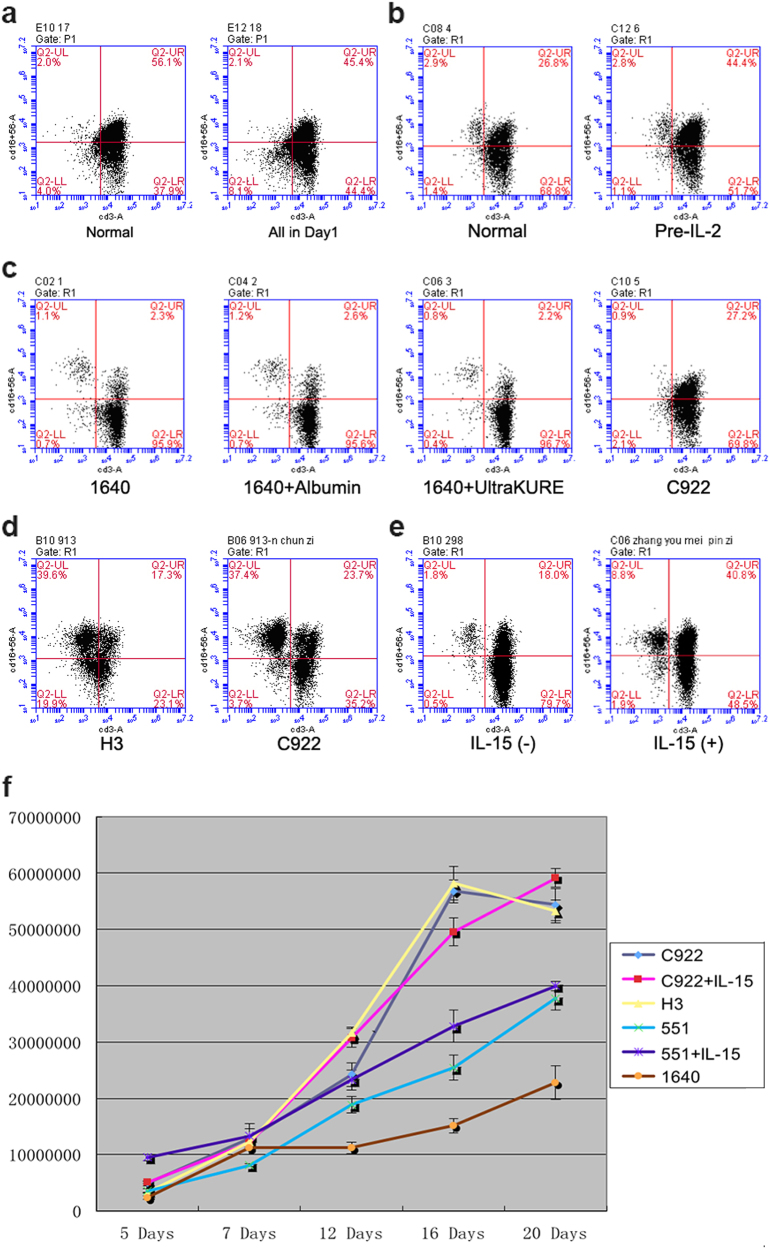
The effect of cytokines on the differentiation and amplification of CIK cells. **a** Different adding orders of stimulus affect the phenotypes of CIK cells. Normal: PBMCs were treated with IFN-γ for 24 h and then supplemented with IL-1α, IL‑2, and CD3 mAb. All in day 1: PBMCs were directly incubated with IFN-γ, IL-1α, IL‑2, and CD3 mAb all in the day 1. Cell phenotypes (CD3^+^CD56^+^) were identified by flow cytometry. **b** Adding orders of IL-2 influence the phenotypes of CIK cells. Normal: the adding orders are the same with **a**. Pre-IL-2: PBMCs were first treated with IFN-γ and small amount of IL-2 (100 U/mL) for 24 h, and then added with IL-1α, IL‑2, and CD3 mAb according to the Methods. **c** Cytokines affect the phenotypes of CIK cells. According to the Methods, PBMCs were cultured in C922 medium or RPMI‑1640 medium or supplemented with albumin (5%) or UltraKURE for 14 days prior to being subjected to flow cytometry analysis. **d** The comparison phenotypes of CIK cells cultured, respectively, in GT-551H3 and C922 medium. According to the Methods, PBMCs were cultured in GT-551H3 or C922 medium, and 14 days later cells were subjected to flow cytometry analysis. **e** The effect of IL-15 on the activation of CIK cells. CIK cells were cultured in C922 medium as described previously and were added IL-15 or not every 2–3 days. Cell phenotypes (CD3^+^CD56^+^) were identified by flow cytometry. **f** The effect of medium and cytokines on the amplification of CIK cells. CIK cells were cultured, respectively, in the described mediums. Cell numbers were conducted using Cellometer at indicated time points

The traditional method for activating and expanding CIKs was that cells were cultured with IFN-γ for the first day, and with CD3, IL-1α, and IL-2 for the second day. Sometimes, the PBMCs were separated by special techniques called hemapheresis, and the cells were incubated with all the stimulus at the first day. The percentage of activated CIKs (CD3^+^CD56^+^) cultured for normal method (56.1%) was higher than that added all in first day (45.4%) (Fig. 2a). The IL-2R expression level on the surface of PBMCs determined the efficiency of CIKs’ activation. Low dose IL-2 could induce the expression of IL-2R, and that could elevate the activation of the CIKs (Fig. 2b).

The commonly used SFM is the RPMI1640 added albumin. In recent years, some knockout serum replacements were added into the 1640 for CIKs in vitro expansion. Cells cultured in SFM (1640, 1640+ albumin, 1640+ UltraKURE) had relative poor expansion (Fig. 2c). C922, a SFM has been developed for in vitro activation and expansion of human T lymphocytes, which enhanced the growth and activation of CIKs (Fig. 2c). Compositions of C922 medium are shown in Supplementary Table 1. The activation status comparison of commercial H3 medium and self-made C922 medium was carried out in three independent experiments. The proportion of activated CIKs (CD3^+^CD56^+^) was higher in the C922, but the NK (CD3^−^CD56^+^) ratio was higher in the H3 (Fig. 2d). It was recently reported that IL-15 stimulation resulted in a significant enhancement of the activation of CIK cells. The percentage of activated CIKs (CD3^+^CD56^+^) was higher than that did not supplement IL-15 (Fig. 2e). The kinetics of proliferation of CIK cells under different culture conditions were shown in Fig. 2f. C922 and H3 medium promoted the more rapid expansion of CIKs compared with others (Fig. 2f and Table 1). Absolute numbers of CIKs obtained in C922 medium together with IL-15 varied from 5.04 × 10^6^ to 5.92 × 10^7^ cells, indicating a mean 18-fold expansion.

**Table 1:** Efficiency of different culture media for CIK proliferation

	C922	C922 + IL-15	H3	551	551 + IL-15	1640
C922		0.423	0.229	***	***	***
C922 + IL-15	0.423		0.670	***	***	***
H3	0.229	0.670		***	***	***
551 + IL-15	***	***	***		***	***
551	***	***	***	***		***
1640	***	***	***	***	***	

Repeated measurement ANOVA was used

***P < 0.001

### The cytotoxic activity of CIK cells on the H1299 cells

**Fig. 3:**
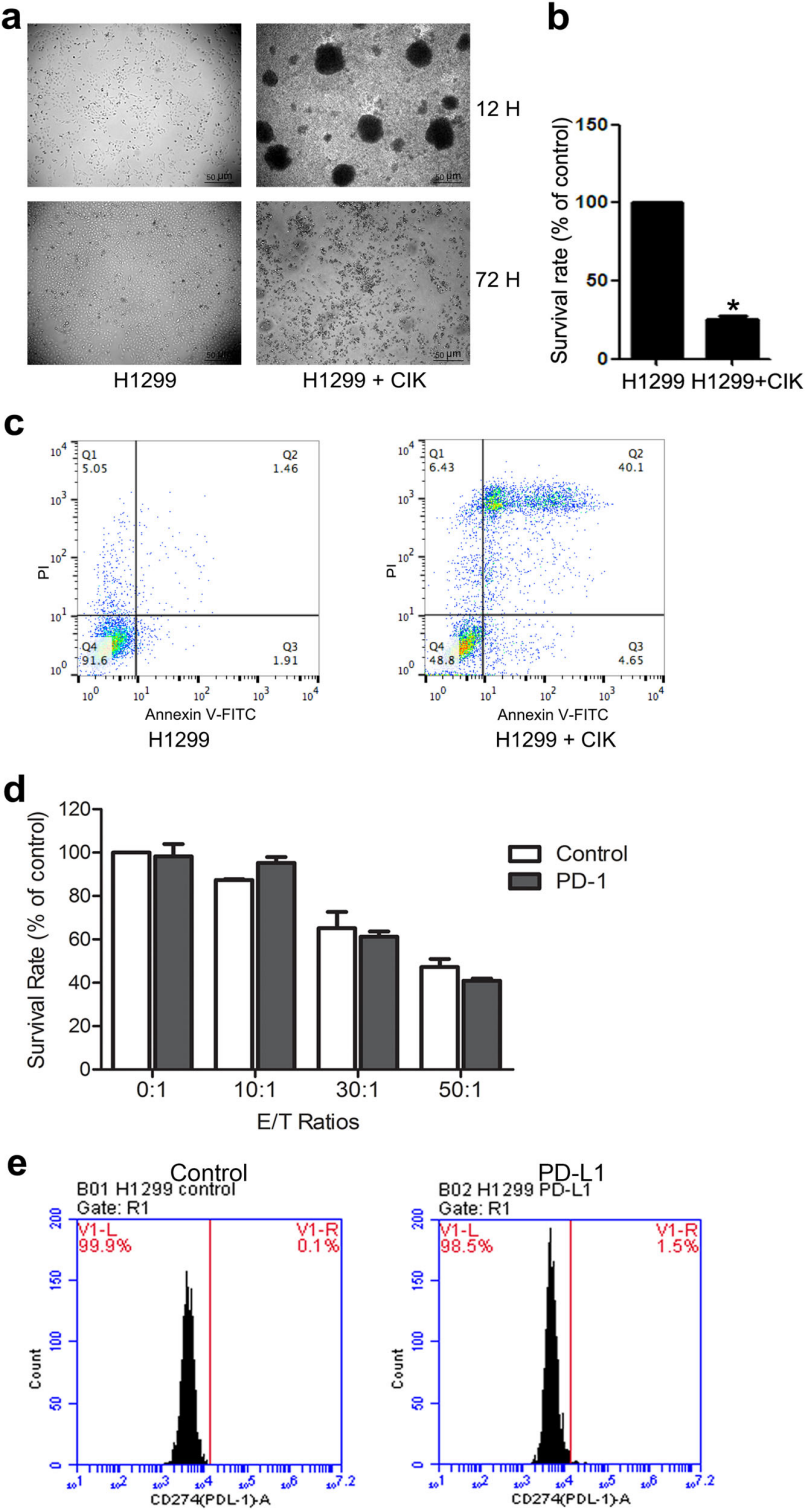
The cytotoxicity of CIKs and PD-1 Abs on H1299. **a** The cytotoxicity of CIKs on H1299 showed in the bright-field images. CIKs were prepared referring to the Methods. H1299 were seeded into 24-well plates, and at ~60% confluence CIKs were added into H1299 at E:T (effector-to-target) ratio of 80:1. H1299 was observed under a microscope after 12 or 72 h of incubation with CIKs (magnification, ×50). **b** Cytotoxicity of CIK cells against H1299 cells. H1299 was co-cultured with CIKs at E:T ratio of 80:1 for 72 h and was subjected to CCK8 assay. Data was obtained from three independent experiments. **c** H1299 was co-cultured with CIKs at E:T ratio of 80:1 for 72 h and was subjected to Annexin V-FITC/PI double-staining apoptosis assay. Data was obtained from three independent experiments. **d** Cytotoxicity of CIK cells combined with PD-1 Abs against H1299 cells. H1299 was co‑cultured with CIKs at various E:T ratios (0:1, 10:1, 30:1, 50:1), and then incubated with PD-1 Abs or not. The cell viability was conducted by CCK8 assay. Data was obtained from three independent experiments. **e** The expression level of PD-L1 in H1299 cells. The expression of surface PD-L1 on H1299 were measured by flow cytometry using CD274 antibody

The cytotoxicity of CIKs was measured after 72 h co-culturing with H1299 and removing suspensions. CIKs clusters exhibited high anti-tumor effect on H1299 cells (Fig. 3a). CIK cells efficiently suppressed the profession and killed H1299 cells with a significant difference (*P* < 0.01) (Fig. 3b, c).

PD-1 pathway induces immunosuppression that plays a negative regulatory role in response to tumor progression^26^. PD-1-specific agonist monoclonal antibody may facilitate maximal cytotoxicity of CIK cells to kill tumor cells. Unfortunately, there was no statistical significance between the CIKs group and the combination of PD-1 mAbs group at various E:T ratios (Fig. 3d). PD-L1 functions as “molecular shield” on cancer cells that prevents effector immune cells from killing cancer cells. The expression level of PD-L1 on H1299 cells was measured by flow cytometry using CD274 antibody. H1299 was the PD-L1-negative cell line on their surface (Fig. 3e).

### The efficiency of CIK cells against NSCLC in nude mice

**Fig. 4:**
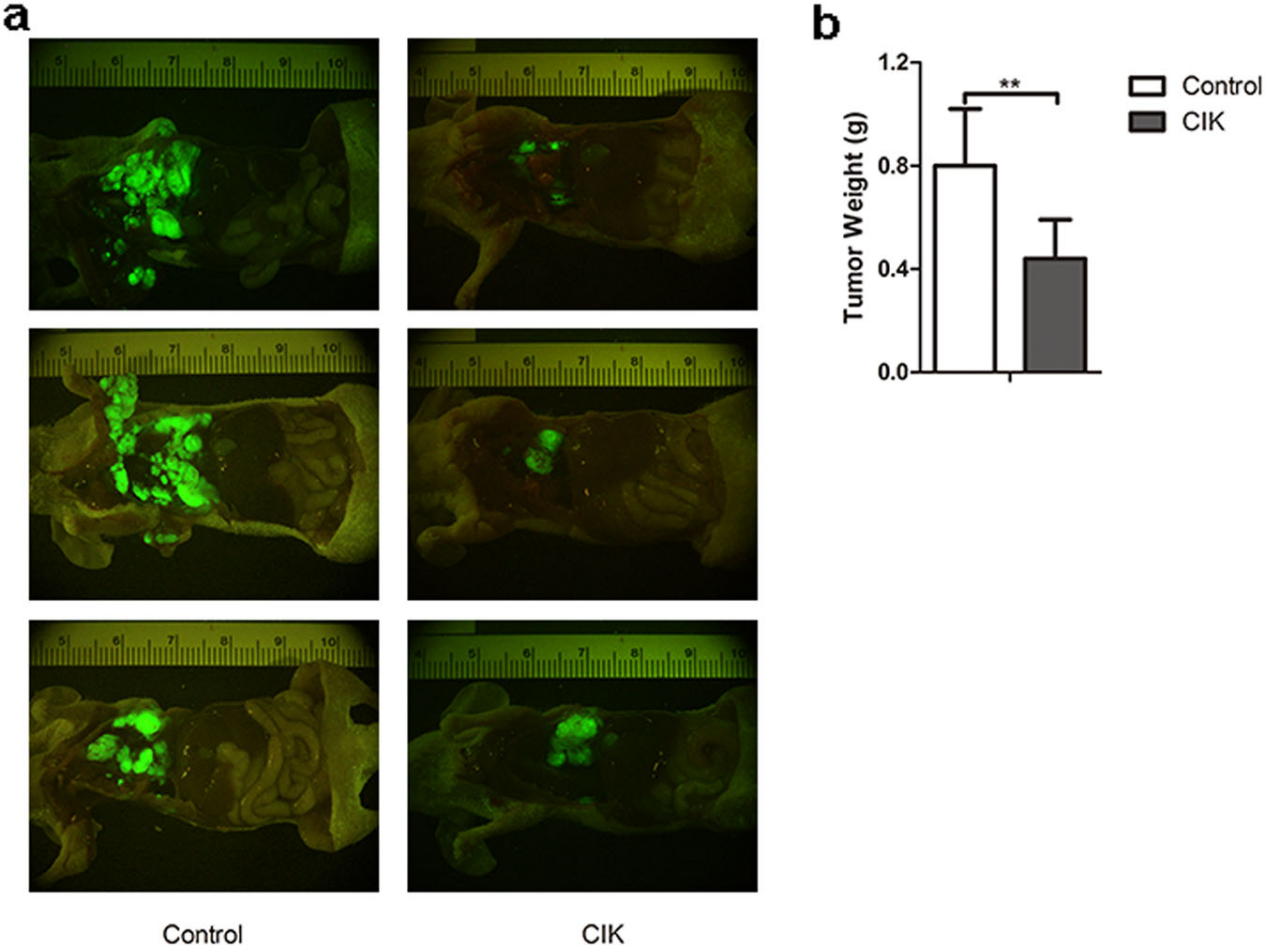
Xenograft experiments. **a** In vivo fluorescence images showing that the size of the tumor mass of mice from the CIK group significantly reduced. **b** Histogram revealing that the average weight of the tumor mass was significantly reduced in the CIK group. Error bars indicate the mean ± SD. **P < 0.01 in *t* test

In order to further investigate the anti-tumor activity of CIKs treatment in vivo, the nude mice were grouped and treated as described above (6 per group). The results revealed that the size of the tumor mass in the CIK group were significantly reduced compared with that in the control group (Fig. 4a), while there was no significant difference between the CIK group and the control group in terms of average weight of the mice (Supplementary Fig. 1). The tumor masses from the two groups were isolated on day 44 after grafted, and the average weights of them were consistent with the fluorescence images (Fig. 4b).

### Demographic data and clinical pathological characteristics

From September 2012 to October 2015, 68 patients with lung cancer underwent chemotherapy combined with CIK cell therapy at our department were included (T group) and another 68 lung cancer patients who received chemotherapy alone were taken as control (C group). No statistical difference was found regarding the characteristics of patients between T group and C group (*P* for all >0.05) (Supplementary Table 2).

### Change of immunophenotype

**Fig. 5:**
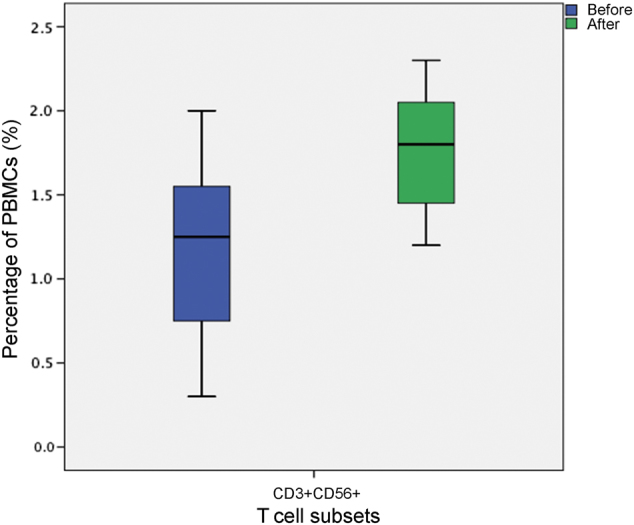
The change of CD3^+^CD56^+^ after CIK treatment

PBMCs from the same patient were obtained before and after CIK cell transfusion. Assessment of immunophenotype phenotypes was determined by flow cytometry. As shown in Table 1 and Fig. 5, after CIK therapy, the index CD3^+^ CD56^+^ (1.05 vs. 1.55) of patients in T group was significantly increased, while no significant differences were observed in percentages including CD3^+^, CD3^+^ CD4^+^, CD3^+^ CD8^+^, CD4/CD8 and CD3^−^CD56^+^ (Table 2 and Supplementary Fig. 2a, b).

**Table 2:** Change of T-cell subsets after CIK treatments

T-cell subsets	Before	After	^a^*P* value
CD3^+^ (%)	61.65 ± 10.94	61.67 ± 11.22	0.991
CD4^+^ (%)	52.64 ± 15.28	50.76 ± 15.01	0.276
CD8^+^ (%)	35.61 ± 12.17	36.10 ± 11.07	0.737
CD4/8	1.81 ± 1.11	1.59 ± 0.89	0.078
CD3^+^CD56^+^ (%)	1.05 ± 0.57	1.55 ± 0.62	0.004
CD3^−^CD56^+^ (%)	18.06 ± 5.69	19.60 ± 7.38	0.286

^a^Paired *t* test was used

### Efficacy of CIK treatment

**Fig. 6:**
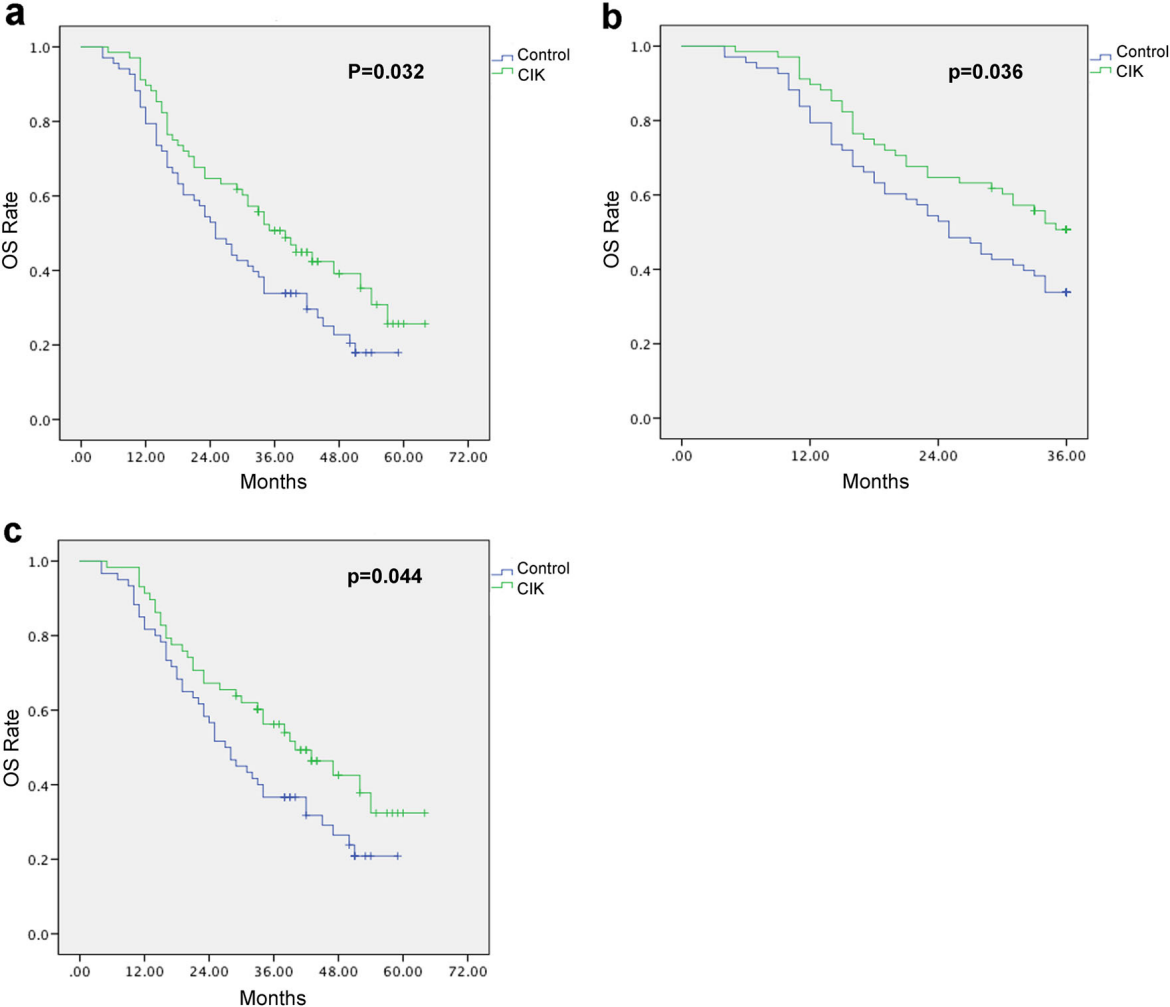
Efficacy of CIK treatment in lung cancer patients. **a** Overall survival (OS) of the patients in the two groups. **b** 3-year OS rate of the patients in the two groups. **c** OS of NSCLC patients in the two groups. Kaplan–Meier method was used to estimate the OS rate and survival curves, *P* values of <0.05 were considered statistically significant

The date of the last follow-up visit was 31 December 2017. As presented in Fig. 6, the median OS curves showed that the median OS of patients in the T group was longer than that in the C group (38 vs. 30 months) with a significant difference, according to log rank analysis (*P* = 0.032) (Fig. 6a). For CIK group, the 3-year OS rate was 50.7, which was significantly improved compared to that in the control group (the 3-year OS rate: 33.8%, *P* = 0.036, Fig. 6b). Moreover, we performed the median OS curves for NSCLC and SCLC separately. The median OS in NSCLC patients was significantly improved in the T group than that in the C group (40 vs. 32 months, *P* = 0.044, Fig. 6c). Although median OS in SCLC patients was 28 months in T group while 19 months in C group, no significant difference was observed (*P* > 0.05, supplementary Fig. 4).

### Adverse reactions during treatment in the two groups

One patient had temporary arrhythmia during chemotherapy in the C group, which was relieved within half an hour after treatment. Comparisons of the incidence of myelosuppression, vomiting, and fever revealed no significant differences between the two groups. There was no severe liver toxicity, renal toxicity, peripheral nerve toxicity, or other fatal adverse reactions in the two groups (Table 3).

**Table 3:** Toxicity and side effects of the two groups

Adverse event	T group		C group		^a^*P* value
Myelosuppression					1.000
I–II degrees	6	8.82%	7	10.94%	
III–IV degrees	5	7.35%	4	5.88%	
Vomitting	1	1.47%	1	1.47%	1.000
Arrhythmia	0	0%	1	1.47%	1.000
Fever	4	5.88%	3	4.41%	1.000

^a^*χ*^2^ test and Fisher exact test were used

## Discussion

The activation and amplification of CIK cells are generated by culturing PBMCs with the timed addition of anti-CD3 antibody and the cytokines IFN-γ, IL-1α, and IL-2 for 14 days^27^. CD3^+^CD56^+^ cells are the distinguishing cell population among the CIK cells and have the most potent cytotoxic function. CD3 mAb is an important stimulus in the activation and proliferation of CIKs^13,28^. As shown in Fig. 1, CIKs could not be activated and amplified efficiently without CD3 mAbs. CD3 antibody from human did not elicit the activation and proliferation of CIKs, but CD3 antibodies of the same clone number (OKT3) from mouse could amplify well. The concentration (100 ng/mL) of CD3 mAbs could adequately activate and amplify CIKs. On the contrary, the high concentration of CD3 mAbs suppressed the activation of CIKs. It means that high concentration of CD3 mAbs cannot elicit more activation. We suspected that high concentration might have cytotoxicity to the cells, which limited its effects. In addition, humanized CD3 antibody was designed to activate and amplify CIKs sufficiently and solve the associated ethical problems.

Few clinical studies have confirmed the role of CIKs in the maintenance therapy of advanced lung cancer patients. CIKs, alone or in combination with other therapies, have proven safe and efficient in clinical practice^22–24,29,30^. CIK cells consist of a heterogeneous cell population, with the primary population comprising CD3^+^ CD56^−^ and CD3^+^ CD56^+^ cells and a minor population of CD3^−^ CD5^+^ cells^13^.

Adoptive cell immunotherapy elicits unprecedented controversial responses in treatment for cancer patients with individual differences, and only small fraction of patients benefit from it^25^. As shown in Fig. 3a–c, the activated CIK cells can efficiently kill some kinds of tumor cells in cell-to-cell model, but it had relatively low response rates in patients, which indicated the cellular immunotherapies were not able to release their full potential in many situations.

Recently clinical trials focused on the tumor site ongoing immune modulation and anti-PD therapy for cancer^26,31^. Some research groups attempted combination of anti-PD therapy, which should facilitate adoptive cell immunotherapy reaching its maximal effect to control tumor growth^32,33^. During activation and amplification of cytotoxic T lymphocytes in vitro, we monitored the expression level of PD-1 on T cells by flow cytometry using CD279 antibody. We found that the level of PD-1 on T cells was rising along with the amplification of T cells (data not show). We used PD-1- specific agonist monoclonal antibody to block PD-1 and measured the cytotoxicity of T cells in vitro. Unfortunately, as shown in the Fig. 3d, there was no statistical significance between the cell therapy and the combination of PD-1 mAbs. some pioneering paper illustrated rare PD-L1 expression on the cell surface of most in vitro–cultured tumor cell lines but abundantly expression on the cell surface in various human cancers^34,35^. We have confirmed that the tumor cell lines, as shown in Fig. 3e, such as H1299 was PD-L1- negative on their surface. This might explain why the combination of PD-1 mAbs had no effect on suppressing the growth of H1299 in vitro. The pattern of PD-L1 expression was found to be clustered rather than diffused in most human cancers, and was generally localized to the area where IFNγ+ T cells infiltrate^34^. Thus, samplings from needle biopsy only reflect small fraction of tumor tissue, and it may miss PD-L1 clustered area and give false negative results. It is difficult to evaluate the clinical response to anti-PD therapy under this circumstance. Next, we will transfect PD-L1 to the tumor cell lines and to create models to study how tumor-associated PD-L1 interacts with immune cells. It will be particularly interesting to see whether or not adoptive cell therapy can be used in combination with anti-PD therapy for treatment of human cancer in vivo.

In our study, our results suggested that, compared to chemotherapy alone, CIK therapy in combination with chemotherapy significantly improved survival time. This was consistent with findings of Zhang et al.^24^ Additionally, CIK therapy represented good toleration in our study. Moreover, several recent papers also explored the role of CIK in lung cancer patients. Luo et al.^23^ demonstrated CIK treatment was safe and effective for advanced lung cancer patients. Li et al.^22^ reported that chemotherapy combined with CIK therapy could improve OS in advanced NSCLC patients compared with chemotherapy alone. Before, during and after cell therapy, the presence of CD3^+^, CD3^+^CD4^+^, CD3^+^CD8^+^, CD4^+^/CD8^+^, CD3^−^CD56^+^, CD3^+^CD56^+^ in PBMCs of patients was monitored, which are associated with immune recovery. However, our results for change of immune cell phenotype showed the percentages of CD3^+^CD56^+^ cells were increased after CIK cell transfusion while others had no obvious change. The sample size of our patients was quite small, which might lead to possibility of selection bias and errors. Thus, it might not be sufficient to make such a conclusion.

In present situation, the patients with advanced and metastatic tumors will never turn to immunotherapy, until they have no response to chemotherapy or targeted therapy. Most of patients choose immunotherapy is the one with advanced malignant tumor. It is difficult for us to evaluate the true effect of adoptive cell immunotherapy on cancer.

## Methods

### Preparation of CIK cells and lung cancer cells

Blood (60–100 mL) were obtained from umbilical cord blood of patients. PBMCs were isolated by Ficoll-hypaque (density was 1.077 g/mL) density-gradient centrifugation, and sometimes the PBMCs were separated by hemapheresis. PBMCs (å 2.0 × 10^6^ per mL) were plated into 25 cm² flask (Corning, #431463) and cultured with GT-T551-H3 (Takara, #WK593S) containing human IFN-γ (1.0 × 10^3^ IU/mL, PeproTech, #300-02). The next day, anti-CD3 monoclonal antibody (Clone OKT3) (100 ng/mL, TaKaRa, #T210) or humanized anti-human CD3 monoclonal antibody (100 ng/mL, T & Biological Technology, #TL101), IL-1α (1.0 × 10^3^ IU/mL, PeproTech, #200-01 A), and IL-2 (1.0 × 10^3^ IU/mL, Jiangsu Kingsley Pharmaceutical Co., Ltd.) were added to the medium. The cells were incubated in a humidified atmosphere with 5% CO_2_ at 37 °C. The medium was changed every 2 or 3 days. The incubation lasted 14 days during which culture, bacteria, fungi, mycoplasma, endotoxin, and cell phenotype (CD3, CD8, CD56) should be detected to ensure the availability of CIK cells.

The human NSCLC cell line NCI-H1299 (#CRL-5803) was purchased from ATCC and was maintained in RPMI-1640 Medium (#A10491-01, Gibco) supplemented with 10% fetal bovine serum (#10100-147, Gibco), added with 100 U/mL penicillin and 100 μg/mL streptomycin (Seromed, Jülich, Germany) and grown at 37 °C in a humidified atmosphere of 5% CO_2_.

### Flow cytometry

The phenotype of CIK cells were identified by flow cytometry. After 14 days’ culture, 10 mL cell suspension was extracted from CIK cell culture bags. Cells were washed with phodphate-buffered saline (PBS, Keygen, # KGB50011) for twice and then were stained with mAbs CD3-FITC, CD56-PE (Becton Dickinson Biosciences, San Jose, CA, USA, #340042). H1299 was stained with mAbs CD274 (Becton Dickinson Biosciences, San Jose, CA, USA, #561787) for detecting the surface level of PD-L1. Cells were incubated with associated Abs for 15 min at room temperature and then were washed with PBS. The prepared cells were analyzed by flow cytometry using FACScan analyzer. Qualified CIKs should meet these following criteria: percentage of CD3^+^CD56^+^ cells (å 15%).

### Cell proliferation assay

Cell proliferation assays were performed by CCK8 (#CK04, Dojindo). For CCK8 assay, H1299 (8 × 10^3^) was plated in quadruplicate into 96-well plates and were incubated for 24 h. Following being treated with different E/T (effector cells/target cells, CIK/H1299) ratios for 36 h, cells were incubated with 10 μL CCK8 per well for 2 h. The absorbance of each well was measured at 450 nm using an EL ×800 Universal Microplate Reader (Bio-Tek, Inc.). The proliferation amount of the control H1299 was indicated as 100%, and results were expressed as relative proliferation.

### Cell death assay

H1299 cells (5 × 105) were seeded in each well of six-well plates and incubated for 24 h. After being treated with CIK cells for 72 h, the each tube was added 500 μL of 1× binding buffer and they stained with 5 μL of Annexin V-FITC and 5 μL of propidium iodide (PI) (Keygen, #KGA105) in the dark for 15 min. Then, they were analyzed with the flow cytometer (FACSVerse/Calibur/AriaII- SORP, BD, America).

### Animal models

Male BALB/c nude mice (5–6 weeks old) were purchased from Shanghai experimental animal center, China Academy of Science (Shanghai, China). Mice were maintained in the animal center of Nanjing Medical University and were housed in cages with an automatically controlled temperature (22 ± 2 °C), relative humidity (50–60%), and light (12 h light/dark cycles). All procedures were approved by the Animal Care and Use Committee of Nanjing Medical University, China. After acclimatization for 1 week, Mice revived 200 μL H1299-G cells suspensions (5 × 10^7^ cells per mL) by subcutaneous injection on the back. After tumor reached about 300–500 mm³, the tumors were peeled off from nude mice completely, and then were cut into 1 mm × 1 mm × 1 mm tissue pieces. A piece of tissue was planted into the left lung. After the size of in situ tumor reached about 60–80 mm³, mice were randomly divided into two groups. Each group was composed of six mice and were injected PBS or CIK (5 × 10^6^) into the veins of the tails. Animal body weight was monitored every 4 days for 5 weeks. When the experiment ended, the mice were record by small animal in vivo imaging spectrometer and the tumors were collected immediately and measured.

### Ethics statement

The study was conducted in accordance with the standards of the Declaration of Helsinki and consistent with current ethical guidelines, which was also approved by Ethic Committee of the Nanjing Medical University (no. 2016 [203]). All patients in our retrospective study provided written informed consent forms before using their medical record data.

### Patient selection

A total of 68 lung cancer patients who received chemotherapy combined with CIK treatment from September 2012 to October 2015 were enrolled into our study (T group). In addition, another 68 lung cancer patients who received chemotherapy alone during the same period were taken as control group (C group). All the participants in this retrospective study were all pathologically diagnosed. All these patients should meet a number of inclusion criteria as follows: (1) age between 18 and 80, (2) a Karnofsky performance status score >60, (3) life expectancy >3 months, (4) no obvious heart, liver, kidney, and pulmonary insufficiency. Data including age, gender, histological type, clinical stage, surgical, radiotherapy, CIK cycles, and survival time are collected. The clinical characteristics of the study population were summarized in supplementary Table 2.

### Treatment plan

All patients included finished platinum-based doublet chemotherapy at least four cycles in our study. Dosages were based on National Comprehensive Cancer Network (NCCN) guidelines. The first-line chemotherapy plans for NSCLC patients included PP (Pemetrexed 500 mg/m^2^ day 1 and Cisplatin 75 mg/m^2^ day 1, or Carboplatin AUC 5 day 1), TP (Paclitaxel 200 mg/m^2^ day 1 and Carboplatin AUC 6 day 1), DP (Docetaxel 75 mg/m^2^ day 1 and Cisplatin 75 mg/m^2^ day 1), GP (Gemcitabine 1250 mg/m^2^ day 1, 8 and Cisplatin 75 mg/m^2^ day 1), or NP (Vinorelbine 25–30 mg/m^2^ day 1, 8 and Cisplatin 75–80 mg/m^2^ day 1). SCLC patients were treated with etoposide 80 mg/m^2^ day 1, 2, 3 and Cisplatin 80 mg/m^2^ day 1 or Irinotecan 60 mg/m^2^ day 1, 8, 15 and Cisplatin 60 mg/m^2^ day 1 as the first-line chemotherapy (Supplementary Fig. 3). Patients in the T group received autologous CIK cell treatment after chemotherapy and the therapy was terminated when the patients refused to do or the disease progression occurred.

### Evaluation of adverse events

Adverse events for each patient were evaluated according to the National Cancer Institute common toxicity criteria (NCICTC, version 3.0).

### Statistical analysis

OS rate and survival curves were conducted by Kaplan–Meier analysis. Differences in baseline characteristics, between the cases and controls were evaluated by the *χ*² test for categorical variables and Student’s *t* test for continuous variables. Paired *t* test was used to evaluate the CIK treatment. Differences of the toxicity and side effects between two groups were evaluated by the *χ*² test and Fisher exact test. We applied repeated measurement analysis of variance to analyze the data of different culture media for the activation and proliferation of CIKs. All statistical analyses were performed by statistical analysis software (SPSS 21.0).

## Electronic supplementary material

Supplementary Table 1

Supplementary Table 2

supplementary Figure 1

supplementary Figure 2

supplementary Figure 3

supplementary Figure 4

Supplementary figure legends
