# Supplementary material for: Cytokine-induced killer cells as a feasible adoptive immunotherapy for the treatment of lung cancer
Source: Cell Death Dis. 2018 Mar 6;9(3):366. doi: 10.1038/s41419-018-0404-5 (PMC5840363; doi:10.1038/s41419-018-0404-5)
Supplement: Supplementary file 1 — Supplementary Table 1 [file 41419_2018_404_MOESM1_ESM.docx]

Table 1

Composition of the C922 medium.

| Composition | Concentration (*/L) |
| --- | --- |
| RPMI 1640  (Basic medium)  NaHCO3  Bovine serum albumin | 2 g  5 g |
| Insulin  D-Glucose  Ferric citrate  Ethanolamine  Linoleic acid  Oleic acid  Palmitic acid  *myo*-Inositol  L-glutamine  L-Phenylalanine  Sodium pyruvate  1-thioglycerol  Cholesterol  Hypoxanthine  Riboflavin  Amino Acids Solution (50×)  Vitamins Solution (100×)  Polyamine Supplement (1000×)  Antioxidant Supplement (1000×) | 20 mg  1.8 g  2 mg  1.22mg  1 mg  1 mg  1 mg  19 mg  584 mg  7 mg  110 mg  5.41 mg  13.2 mg  5 mg  0.05 mg  20 mL  10 mL  1 mL  1 mL |
|  |  |
