# Supplementary material for: Cytokine-induced killer cells as a feasible adoptive immunotherapy for the treatment of lung cancer
Source: Cell Death Dis. 2018 Mar 6;9(3):366. doi: 10.1038/s41419-018-0404-5 (PMC5840363; doi:10.1038/s41419-018-0404-5)
Supplement: Supplementary file 2 — Supplementary Table 2 [file 41419_2018_404_MOESM2_ESM.docx]

Table 2

Baseline characteristics of included patients.

| Characteristics | T group (N=68) | C group (N=68) | *P^a^* value |
| --- | --- | --- | --- |
| Age (Mean± SD)  Gender  Male  Female  Histological type  Adenocarcinoma  Squamous cell carcinoma  sclc  Clinical stage  Ⅰ  Ⅱ  Ⅲ  Ⅳ  Surgical  Yes  No  Radiotherapy  Yes  No  CIK cycle  1  2  3  4  5  8 | 61.24 ± 9.82  53 77.94%  15 22.06%  45 66.18%  13 19.12%  10 14.71%  5 7.35%  9 13.24%  17 25.00%  37 54.41%  19 27.94 %  49 72.06%  23 33.82%  45 66.18%  34 50.00%  18 26.47%  9 13.24%  3 4.41%  3 4.41%  1 1.47% | 59.57 ± 10.12  50 73.53%  18 26.47%    45 66.18%  15 22.06%  8 11.76%  6 8.82%  6 8.82%  14 20.59%  42 61.76%  20 29.41%  48 70.59%  18 26.47%  50 73.53% | 0.333  0.548  0.833  0.730  0.850  0.350 |
|  |  |  |  |

^a^ Student’s *t* test and *χ2* test were used for continuous and categorical variables, respectively.
