# Supplementary figures and images for: Cytokine-induced killer cells as a feasible adoptive immunotherapy for the treatment of lung cancer
Source: Cell Death Dis. 2018 Mar 6;9(3):366. doi: 10.1038/s41419-018-0404-5 (PMC5840363; doi:10.1038/s41419-018-0404-5)

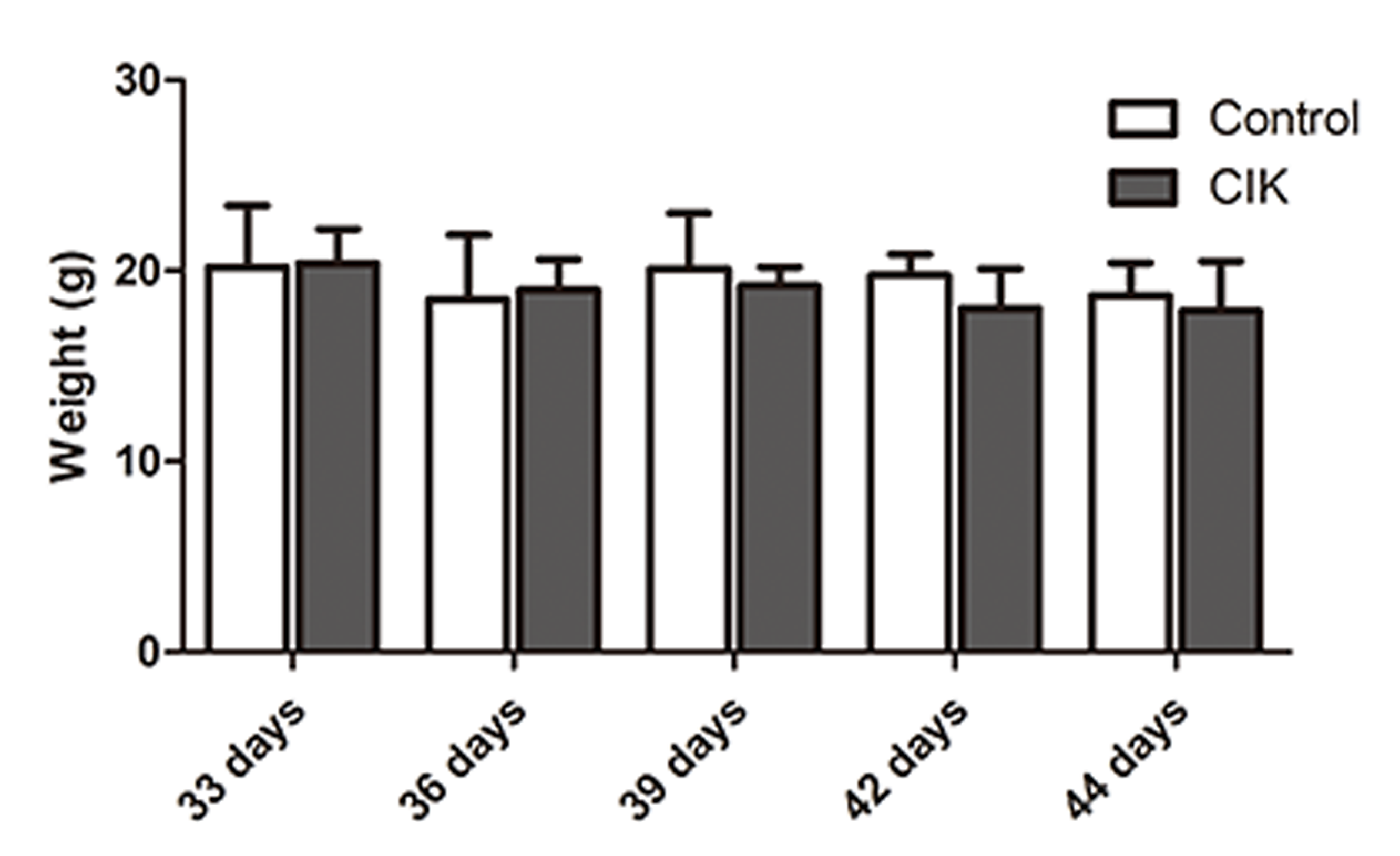

Supplement: Supplementary file 3 — supplementary Figure 1 [file 41419_2018_404_MOESM3_ESM.tif]

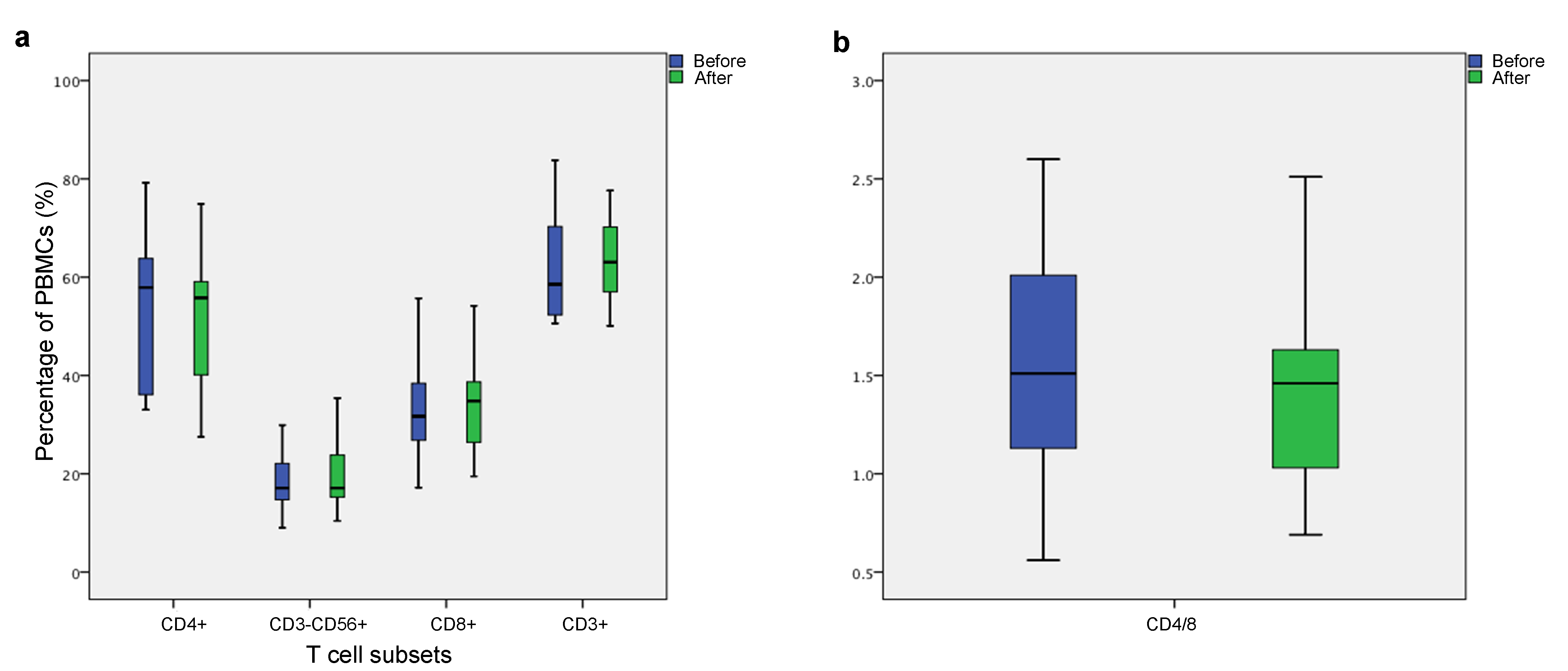

Supplement: Supplementary file 4 — supplementary Figure 2 [file 41419_2018_404_MOESM4_ESM.tif]

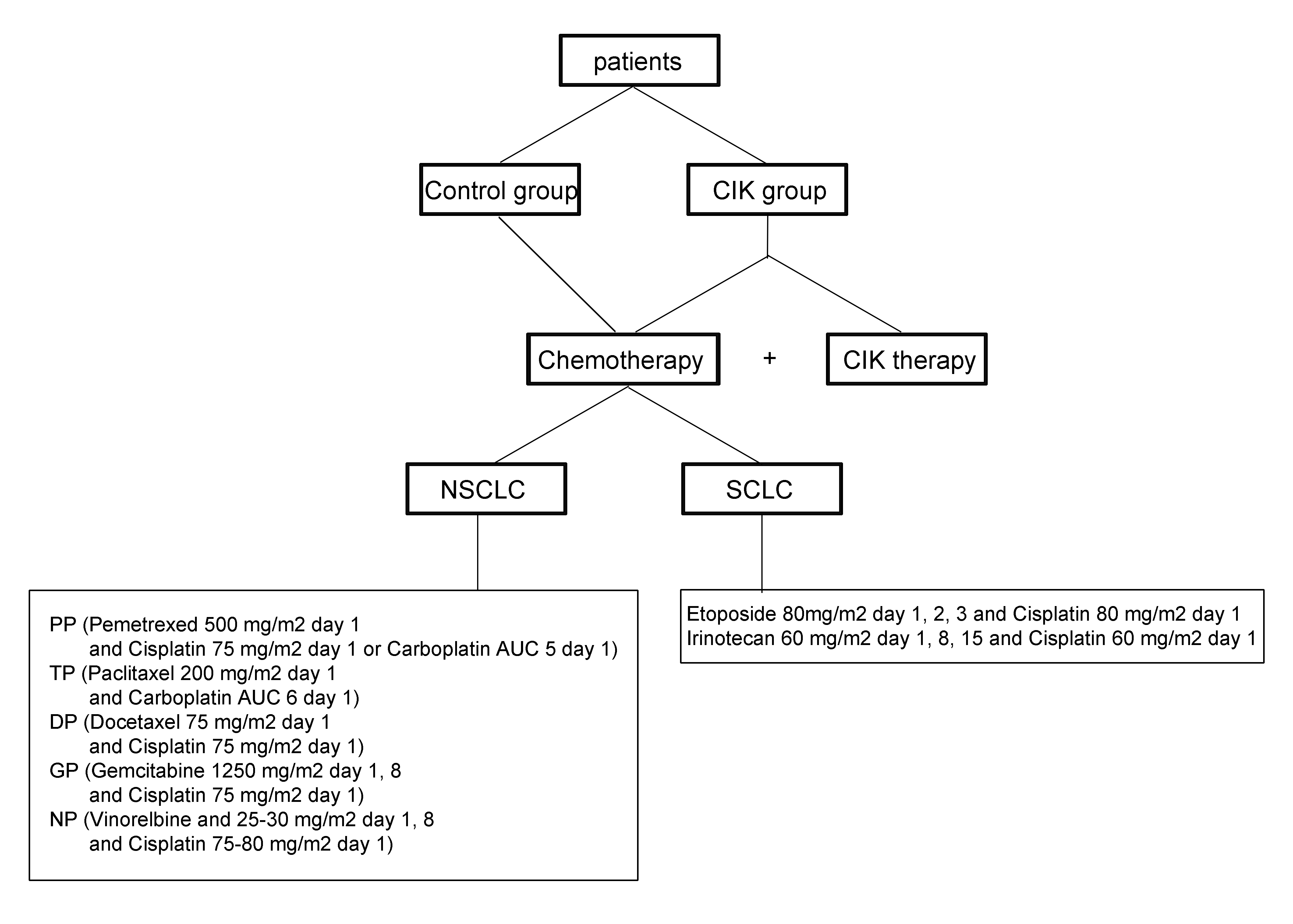

Supplement: Supplementary file 5 — supplementary Figure 3 [file 41419_2018_404_MOESM5_ESM.tif]

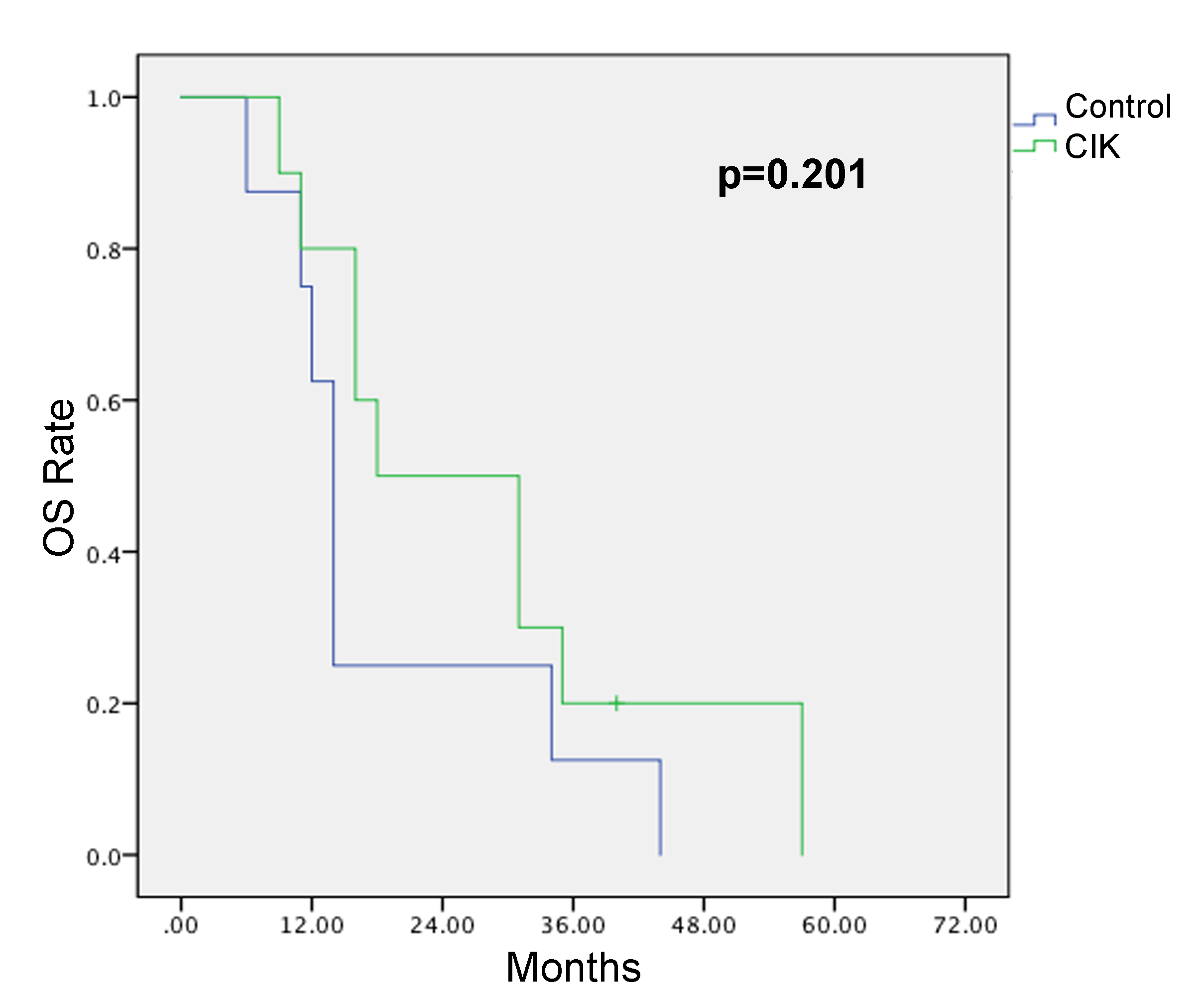

Supplement: Supplementary file 6 — supplementary Figure 4 [file 41419_2018_404_MOESM6_ESM.tif]
