# Supplementary material for: Cytokine-induced killer cells as a feasible adoptive immunotherapy for the treatment of lung cancer
Source: Cell Death Dis. 2018 Mar 6;9(3):366. doi: 10.1038/s41419-018-0404-5 (PMC5840363; doi:10.1038/s41419-018-0404-5)
Supplement: Supplementary file 7 — Supplementary figure legends [file 41419_2018_404_MOESM7_ESM.docx]

Supplementary Fig. 1 Histogram showing no difference in the average weight of the mice in the control group or CIK group. Each experimental group was composed of 6 mice. Error bars indicate the mean ±SD.

Supplementary Fig. 2 Changes of T cell subsets after CIK treatments. (a) Box and whisker plots showed the change of percentages including CD3+, CD3+CD4+, CD3+CD8+ and CD3-CD56+. (b) Box and whisker plots displayed the change of CD4/CD8.

Supplementary Fig. 3 The treatment plan for both the control group and CIK group.

Supplementary Fig. 4 OS of SCLC patients in the two groups. Kaplan-Meier method was used to estimate the OS rate and survival curves, P-value of <0.05 was considered statistically significant.
